# Nomogram for predicting the distal adding-on phenomenon in severe and rigid scoliosis

**DOI:** 10.3389/fsurg.2022.1065189

**Published:** 2023-01-06

**Authors:** Zhongyang Li, Huiliang Yang, Chunguang Zhou, Peng Xiu, Xi Yang, Lei Wang, Ganjun Feng, Limin Liu, Yueming Song

**Affiliations:** Department of Orthopedic Surgery and Orthopedic Research Institute, West China Hospital, Sichuan University, Chengdu, China

**Keywords:** distal adding-on, nomogram, internal distraction, severe and rigid scoliosis, scoliosis

## Abstract

**Background:**

The distal adding-on phenomenon has attracted extensive discussion in the field of spine surgery due to the continual occurrence after scoliosis correction. Previous work has mainly focused on adolescent idiopathic scoliosis (AIS), and a relatively high number of theories for the mechanism of the distal adding-on phenomenon has been proposed for these kinds of patients. Severe and rigid scoliosis, as a special disease form, has a unique etiology, clinical manifestations and internal mechanisms distinct from those of AIS. Given the uniqueness of this disease, the mechanism and causes of the distal adding-on phenomenon have been infrequently studied in depth.

**Objective:**

To define clinical and radiological factors associated with distal adding-on in patients with severe and rigid scoliosis.

**Methods:**

Radiographic parameters and demographic data of patients with severe and rigid scoliosis were evaluated preoperatively, after posterior instrumentation and fusion surgery, and at the final follow-up *via* radiographs. According to the appearance of distal adding-on at the final follow-up, the patients were grouped into the Adding-on and the Non-adding-on groups. Various radiological parameters were analyzed in stepwise multivariate logistic regression to identify the variables associated with distal adding-on, which were then incorporated into a nomogram. The predictive performance and calibration of the nomograms for distal adding-on were assessed using C statistics and calibration plots.

**Results:**

93 patients (21 in the Adding-on and 72 in the Non-adding-on group) were included. The incidence of distal adding-on was 22.6%. The variables associated with distal adding-on were the anterior release, posterior internal distraction, and later posterior spinal fusion (IP) procedure, the posterior vertebral column resection and posterior spinal fusion (PVCR) procedure, postoperative apical vertebral translation (Post-AVT) and preoperative slope of the line linking the pedicles on the concave side of the upper- and lower-end vertebrae (Tan *α*). Combining these factors, the nomogram achieved a concordance index of 0.92 in predicting distal adding-on and had well-fitted calibration curves.

**Conclusions:**

For patient with a negative Tanα in severe and rigid scoliosis, the risk of distal adding-on tended to increase, and it is recommended to give priority to IP or PVCR. In the final correction, a smaller Post-AVT should not be pursued excessively.

## Introduction

Severe and rigid scoliosis is a special disease form of scoliosis with a unique etiology, clinical manifestations and internal mechanisms, presenting as a complex and progressive spinal deformity with many distinctive features. It is generally recognized that patients with severe and rigid scoliosis possess a main curve of more than 80° on normal radiographs and the flexibility of less than 30% on bending radiographs (1, 2).

The distal adding-on phenomenon has attracted extensive discussion in the field of spine surgery due to the continual occurrence after scoliosis correction. It is defined as a progressive increase of the number of vertebrae contained distally within the main curve linked with either an increase in the deviation of the first vertebra below instrumentation from the center sacral vertical line (CSVL) of more than 5 mm or an increase in the angulation of the first disc of more than 5° at a minimum 2-year follow-up (3).

Previous work on the distal adding-on phenomenon has mainly focused on AIS patients, and a relatively high number of theories have been proposed for its mechanism in these kinds of patients (4, 5). Given the unique presentation of severe and rigid scoliosis, however, the mechanism and causes of the distal adding-on phenomenon are rarely studied in depth. Consequently, the objective of the present study was to define clinical and radiological factors associated with distal adding-on in severe and rigid scoliosis using patient data from a single center. Especially, we tried to create and internally validate a nomogram to predict the distal adding-on phenomenon in this disease.

## Patients and methods

### Patients

This study was approved by the ethics committee of West China Hospital of Sichuan University and all patients signed the informed consent. This study was conducted in strict accordance with relevant guidelines and regulations. A review was performed on all patients retrospectively with severe and rigid thoracic or thoracolumbar scoliosis who underwent anterior release and posterior spinal fusion (APSF), anterior release, posterior internal distraction, and later posterior spinal fusion (IP), and the posterior vertebral column resection and posterior spinal fusion (PVCR) at our medical center from January 2008 to June 2018. The inclusion criteria were as follows: (1) main thoracic or thoracolumbar curve greater than 80° and flexibility of less than 30% on bending radiographs, (2) a minimum of two years of follow-up, and (3) no history of spine surgery. The distal adding-on was defined as (1) lowest instrumented vertebra (LIV)-central sacral vertical line (CSVL) > 10 mm, (2) LIV + 1-CSVL > 5 mm, and (3) disc angle of LIV + 1 > 5°. Patients who met at least one criterion were included in the Adding-on group. Altogether, 93 patients (21 in the Adding-on group and 72 in the Non-adding-on group) were included in the present study. General information, including sex, age, and Risser classification, was collected from the patients. Radiographic assessment was performed using full-length anteroposterior (AP) lateral radiographs and passive lateral bending radiographs acquired preoperatively, postoperatively, and at the final follow-up.

### Surgical procedures

All procedures were performed by two senior spine surgeons (L.M.L. and Y.M.S.), and somatosensory evoked potentials and motor evoked potentials were performed. Patients received 3 kinds of surgery (anterior release and posterior spinal fusion (APSF); anterior release, posterior internal distraction, and later posterior spinal fusion (IP); posterior vertebral column resection and spinal fusion (PVCR). The APSF consisted of anterior approach surgery and later posterior spinal fusion. The anterior release involves a thoracic incision and the convex side of the area was resected. The patients were taken to lateral position with the main curve upward to undergo the anterior release. Thoroughly remove the intervertebral disc and the fibrous connective tissue to ensure adequate loosening of the spine. And then expose and remove the rib adhere to the uppermost level of the spine to be approached (6). After anterior release, the patient was converted to prone position for posterior spinal fusion.

For the IP procedure, after anterior release, the patients were turned to prone position for posterior internal distraction. Make two small incisions to expose the vertebral body at the caudal and caudal to be fixed. Subperiosteal dissection was performed to expose the facet and transverse process on the concave side of the spine. To select at least 2 fixing points at the caudal and caudal end respectively to ensure fully fixation. After implanting pedicle screws at the fixed point, 2 pre-bent titanium rods were selected. The long distraction rod passed subcutaneously and connected to the pedicle screw at the cranial end and the short distraction rod was connected with the pedicle screw at the caudal end. Then, connect the two rods through the domino connector (Medtronic, Fort Worth, TX) and lock the pedicle screw cap. After locking the screw caps on the medial side of the domino connector, distraction between the distal pedicle screw and the domino connector was performed. Next, to lock the screw caps on the lateral side and loosen the screw caps on the medial side of the domino connector. Perform a similar distraction between the domino connector and the rod holder (7). During the distraction process, the abnormal evoked potential should be avoided as much as possible. During each distraction, we allow a few minutes of stress relaxation between multiple distractions. One to four weeks after traction, posterior spinal fusion was performed.

For the PVCR procedure, after the general anesthesia, the patients were turned to a prone position with the autologous blood transfusion. Along the spinous process of the predetermined fusion segment, an arc-shaped incision was used to open the skin and subcutaneous tissue. The paraspinal muscles were stripped along the spinous process to the subperiosteal sides on both sides, the supraspinous ligaments of the upper and lower vertebrae of the scheduled fusion levels are preserved, and the lamina and the upper and lower articular processes of the scheduled fusion levels were completely exposed. In the process of screw placement, the lower articular process of the corresponding vertebral body was excised at the same time, and the cartilage surface of the upper articular process was scraped off to obtain adequate release of the spine and facilitating the fusion. Except for the predetermined osteotomy segment, pedicle screws were implanted. After the screw placement completed, C-arm fluoroscopy was performed to check the internal fixation. After the pedicle placement, the spinous process, lamina, bilateral articular process and transverse process of the vertebral body scheduled for osteotomy was excised, and the spinous process, lamina and bilateral articular process of the upper and lower 1–2 vertebral bodies were removed to achieve decompression of the spinal cord and nerve roots., the convex side osteotomy of the vertebral body was performed first followed by the decompression. After resection of the vertebral body, the osteotomy surface and spinal nerve were carefully checked. After the correction operation, the C-arm fluoroscopy was performed to confirm that the implant was stable and in a good position.

### Radiographic evaluation

Double-blind parameter measurements were performed by two experienced spine surgeons, and the measured parameters were averaged as the final result. The Adding-on and Non-adding-on groups were compared according to these variables: operation time, screw number, fusion length, estimated blood loss, follow-up duration, and radiographic parameters. Radiographic parameters included the primary curve, cranial curve, caudal curve, thoracic kyphosis, lumbar lordosis, thoracic apical vertebral translation (AVT), coronal balance (CB), clavicular angle (CA), coracoid height difference (CHD), clavicle-rib intersection difference (CRID), radiographic shoulder height (RSH), sagittal vertical axis (SVA) and slope of the line connecting the pedicles on the concave side of the upper and lower-end vertebrae (Tan *α*). To evaluate CB, draw a vertical plumb line from the midpoint of the C7 vertebral body to measure the horizontal distance between the plumb line and the midline of the sacrum. The SVA was determined by measuring the horizontal distance of the C7 plumb line to the posterior superior corner of the sacrum. The CA was determined by measuring the angle between the lines connecting the highest point of the clavicle on the horizontal plane. The CHD was determined by measuring the difference in height between horizontal lines passing through the upper edge of each coracoid process; negative values indicate right shoulder elevation, while positive values indicate left shoulder elevation. The CRID was determined by the height difference between the horizontal lines passing through the intersection of the upper edge of the clavicle and the outer edge of the second rib on both sides. The RSH was determined by the difference of soft tissue shadows directly above the acromioclavicular joint on standing anteroposterior films. The absolute values were used to check any deviation from the normal value, regardless of the direction of the shoulder (8–10). To assess Tan *α*, a line linking the pedicles on the concave side of the upper- and lower-end vertebrae was drawn, and the tangent of the angle between this line and the vertical plumb line was calculated.

### Statistical analysis

Continuous variables are represented as the mean (SD) and were compared using an unpaired, 2-tailed *t* test. The categorical variables were compared through the χ2 or Fisher exact test. The significance of each variable in the training cohort was evaluated by univariate logistic regression analysis to investigate the independent risk factors of distal adding-on. Variables significantly associated with distal adding-on were included for the stepwise multivariate analysis. Based on the results of multivariable logistic regression analysis, the nomogram was developed through the rms R package, version 3.0 (http://www.r-project.org/). The nomograph is based on scaling each regression coefficient in the multiple logistic regression into a score of 0–100 points. The effect of the variable with the highest *β* coefficient is designated as 100 points. Add these points to get the total number of points, and then convert it into a prediction probability.

For the clinical use of the model, the total scores of each patient were calculated according to the nomogram. Receiver operating characteristic (ROC) curve analysis was used to get the optimal cutoff values, which were depended on maximizing the Youden index (i.e., sensitivity + specificity−1). The accuracy of the best cutoff value was assessed with the sensitivity, specificity, predictive values, and likelihood ratios. In the univariate analyses, *p* < 0.05 was considered to be significant statistically. In the stepwise multivariate analysis, *p* < 0.1 was considered to have a trend statistically. All of the analyses were performed by SAS, version 9.1 (SAS Institute Inc.) and R, version 3.0 (11, 12).

## Results

Altogether, 93 patients (21 in the Adding-on and 72 in the Non-adding-on group) were included in this study. The incidence of distal adding-on was 22.6% in the study. The mean age at surgery in the Adding-on group and the Non-adding-on group was 17.6 ± 3.9 years and 17.7 ± 3.9 years, respectively. The Risser grades in the Adding-on and Non-adding-on group were 3.2 ± 0.7 and 3.4 ± 0.9, respectively, and no significant difference was found. There was no significant difference in operation time, fusion length, screw number, estimated blood loss, or follow-up duration between the two groups. There was no significant difference in the main curve preoperatively, postoperatively, or at the final follow-up between the two groups (Table 1).

**Table 1:** Patient characteristics and outcomes between the two groups.

	Adding- on	Non- adding-on	*p* value*
No. of patient	21	72	
Age (yr)	17.6 (13–28)	17.7 (8–25)	0.87
Gender			
Male	12	42	0.59
Female	9	30	
Risser grade	3.2 (1.5–4)	3.4 (0–4)	0.31
Flexibility (%)			
Main curve	14.1 (4–23)	15.1 (4–27)	0.54
Cranial compensatory curve	32.9 (8–68)	15.9 (4–71)	0.02*
Caudal compensatory curve	35.9 (17–47)	42.5 (2–75)	0.23
Direction of main curve			
Left	6	15	0.32
Right	15	57	
No. of screw	14.2 (12–16)	13.6 (12–16)	0.32
No. of fused level	13.5 (12–15)	14.2 (13–15)	0.45
Op. time (min)	392 (417–515)	453 (390–515)	0.72
Estimated blood loss (ml)	1,182 (765–1,432)	1,092 (800–1,650)	0.78
Follow-up (mon)	39.2 (25–76)	42.5 (24–80)	0.82
Operation			
APSF	11	10	
IP	4	41	0.00
PVCR	6	21	
Etiology			
IS	19	58	
SMS	2	12	0.40
CS	0	2	

APSF, Anterior release and posterior spinal fusion; IP, anterior release, posterior internal distraction, and subsequent posterior spinal fusion; PVCR, the posterior vertebral column resection and posterior spinal fusion; IS, Idiopathic scoliosis; SMS, syringomyelia-associated scoliosis; CS, Congenital scoliosis.

*p < 0.05

The characteristics of the primary thoracic curve, including the Cobb angle and flexibility, were similar in both groups. The Adding-on group had a stiffer cranial compensatory curve preoperatively (*p* < 0.05). In the Adding-on and the Non-adding-on group, the average angles of the caudal compensatory curve were 26.9° ± 12.4° and 20.1° ± 10.1°, respectively (*p* = 0.01). At the final follow-up, the average angles of the caudal compensatory curves were 26.2° ± 13.0° and 18.1° ± 9.9°, and a significant difference was found (*p* = 0.01). No significant difference in CB between the two groups either preoperatively or postoperatively was found. At the final follow-up, the average CB in the two groups was 20.8 ± 16.8 mm and 13.2 ± 13.5 mm, respectively (*p* = 0.04). In the SVA, there was no significant difference between the two groups preoperatively, postoperatively or at the final follow-up. Preoperatively, the average CA was 4.6 ± 2.9 mm and 2.9 ± 3.1 mm in the Adding-on and the Non-adding-on groups, respectively, and a significant difference was found (*p* = 0.03). In the CA, no significant difference was found postoperatively or at the final follow-up. The CHD, the CRID and the RSH preoperatively, postoperatively, or at the final follow-up. There was no significant difference in the incidence of thoracic kyphosis or lumbar lordosis. The average Post-AVT in the Adding-on and the Non-adding-on group were 17.8 ± 9.2 mm and 23.9 ± 12.5 mm, respectively (*p* = 0.04). At the final follow-up, the average Post-AVT was 15.5 ± 9.5 mm and 26.6 ± 13.2 mm, respectively (*p* = 0.00). Preoperatively, the average Tan *α* in the Adding-on group and the Non-adding-on group was −0.11 ± 0.28 and 0.01 ± 0.24, respectively (*p* = 0.04).

The LIV-last-touched vertebra (LTV) averaged 0.9 ± 0.8 levels and 1.1 ± 1.2 levels, and the LIV-last substantially touching vertebra (LSTV) averaged 0.6 ± 1.0 levels and 1.0 ± 1.1 levels in the Adding-on and the Non-adding-on group, respectively, with no significant differences. The LIV-lower-end vertebra (LEV) averaged 2.1 ± 0.9 levels and 2.5 ± 0.5 levels, the LIV-neutral vertebra (NV) averaged −1.3 ± 1.0 levels and −0.4 ± 0.7 levels, and the LIV-stable vertebra (SV) averaged −0.4 ± 1.1 levels and −0.6 ± 0.5 levels in the Adding-on and the Non-adding-on group, respectively, with no significant difference (Table 2).

**Table 2:** Radiographic parameters in the two groups.

	Adding-on	Non-adding on	*p* value
Main curve (deg)			
Pre-operative	98.1 (85–120)	104.3 (85–135)	0.06
Post-operative	37.5 (23–60)	39.1 (24–48)	0.46
Follow-up	39.6 (20–58)	40.2 (20–54)	0.58
Loss of correction	2.1 (−6–12)	1.4 (−10–14)	0.71
Cranial compensatory curve (deg)			
Pre-operative	42.8 (15–64)	49.2 (20–72)	0.10
Post-operative	24.4 (10–47)	29.3 (3–53)	0.11
Follow-up	23.9 (8–38)	27.4 (0–47)	0.22
Caudal compensatory curve (deg)			
Pre-operative	49.0 (29–74)	50.3 (21–80)	0.71
Post-operative	26.9 (5–48)	20.1 (5–47)	0.01*
Follow-up	26.2 (5–47)	18.1 (5–52)	0.01*
Thoracic AVT (mm)			
Pre-operative	77.4 (70–90)	80.2 (50–100)	0.30
Post-operative	17.8 (5–40)	23.9 (5–40)	0.04*
Follow-up	15.5 (5–40)	26.6 (10–45)	0.00*
Thoracic kyphosis (T5-T12) (deg)			
Pre-operative	53.3 (24–90)	50.7 (10–115)	0.68
Post-operative	28.2 (18–40)	31.6 (10–60)	0.18
Follow-up	28.8 (18–51)	29.6 (10–58)	0.77
Lumbar lordosis (T12-S1) (deg)			
Pre-operative	57.8 (49–74)	59.6 (31–90)	0.72
Post-operative	45.1 (31–59)	44.3 (8–62)	0.78
Follow-up	45.3 (36–54)	45.2 (5–69)	0.97
Coronal balance (mm)			
Pre-operative	11.2 (0–50)	13.8 (0–50)	0.45
Post-operative	17.9 (0–42)	13.3 (0–60)	0.20
Follow-up	20.8 (0–38)	13.2 (0–43)	0.04*
Sagittal vertical axis (mm)			
Pre-operative	24.8 (0–70)	18.5 (0–65)	0.18
Post-operative	21.9 (0–80)	14.8 (0–60)	0.09
Follow-up	14.8 (0–30)	19.9 (0–70)	0.22
CA (deg)			
Pre-operative	4.6 (0–9)	2.9 (0–14)	0.03*
Post-operative	4.8 (0–15)	3.4 (0–9)	0.10
Follow-up	3.6 (0–5)	3.2 (0–10)	0.52
CHD (mm)			
Pre-operative	12.1 (0–28)	8.7 (0–45)	0.17
Post-operative	11.7 (0–28)	12.8 (0–32)	0.63
Follow-up	7.3 (0–20)	9.6 (0–30)	0.27
CRID (mm)			
Pre-operative	3.9 (0–13)	4.0 (0–15)	0.91
Post-operative	3.4 (0–18)	3.9 (0–13)	0.49
Follow-up	4.1 (0–12)	4.2 (0–13)	0.96
RSH (mm)			
Pre-operative	9.6 (0–29)	8.1 (0–30)	0.52
Post-operative	11.9 (0–32)	10.0 (0–28)	0.40
Follow-up	9.1 (4–30)	7.9 (4–25)	0.43
Tan *α* (deg)	−0.11 (−0.55–0.28)	0.01 (−0.67–0.31)	0.04*
LIV-LTV (level)	0.9 (0–2)	1.1 (0–3)	0.62
LIV-LSTV (level)	0.6 (−1–2)	1.0 (0–3)	0.39
LIV-LEV (level)	2.1 (1–3)	2.5 (1–3)	0.32
LIV-NV (level)	−1.3 (−3–0)	−0.4 (−1–2)	0.07
LIV-SV (level)	−0.4 (−2–1)	−0.6 (−1–0)	0.68

AVT, apical vertebra translation; CA, Clavicular angle; CHD, Coracoid height difference; CRID, Clavicle-rib cage intersection difference; RSH, Radiographic shoulder height; Tan α, the slope of the line connecting the pedicles on the concave side of the upper and lower end vertebrae; LIV, lower instrumented vertebra; LTV, last touching vertebra; LSTV, last substantially touching vertebra; LEV, lower end vertebra; NV, neutral vertebra; SV, stable vertebra.

*p < 0.05

**Figure 1:**
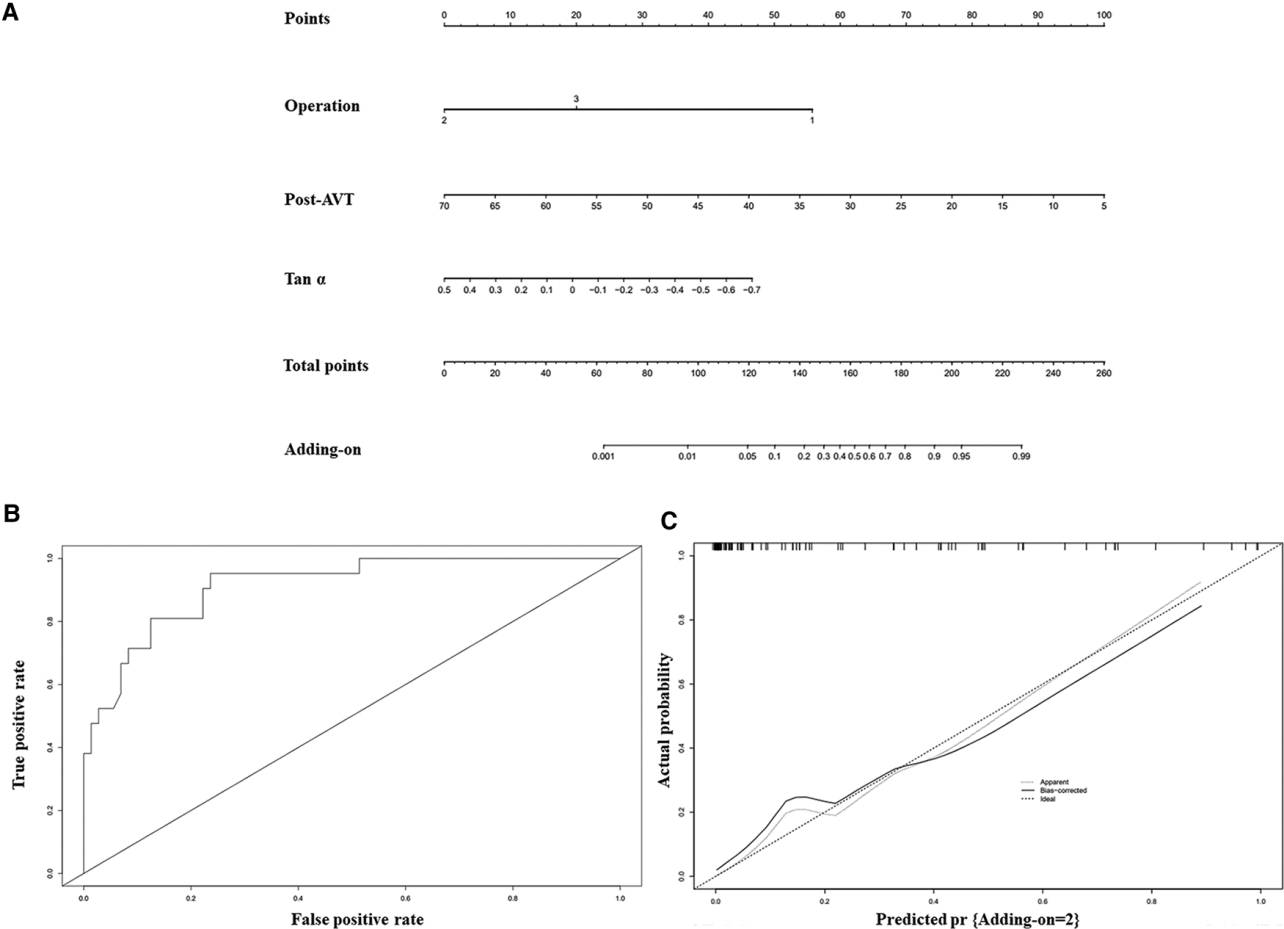
A nomogram for predicting the adding-on phenomenon was created based on independent prognostic factors (**A**). To use the nomogram, find the position of each variable on the corresponding axis, draw a line to the points axis for the number of points, add the points from all of the variables, and draw a line from the total points axis to determine the probabilities of the adding-on phenomenon. The operation 1, 2 and 3 represent APSF, IP, and PVCR respectively. The ROC curve (area under the curve 0.92) (**B**) and the calibration (**C**) indicate that the model performs well.

Established risk factors, as well as clinical and radiological characteristics of adding-on, were selected as candidate variables for the prediction model. The variables associated with distal adding-on included the operation, including IP (OR: 0.02; 95% CI: 0–0.18; *p* = 0.001) and PVCR (OR: 0.08; 95% CI: 0.01–0.56; *p* = 0.011). Post-AVT (OR: 0.9; 95% CI: 0.83–0.97; *p* = 0.008) and Tan *α* (OR: 0.07; 95% CI: 0–1.45; *p* = 0.085) were additional related factors (Table 3). Combining these factors, the nomogram achieved a concordance index of 0.92 in predicting distal adding-on and had well-fitted calibration curves (Figure 1).

**Table 3:** Independent prognostic factors of distal adding-on.

Characteristics	SE	OR	CI	p*
IP	1.12	0.02	0–0.18	0.001*
PVCR	0.98	0.08	0.01–0.56	0.011*
Post-AVT	0.04	0.90	0.83–0.97	0.008*
Tan α (deg)	1.57	0.07	0–1.45	0.085*

IP, anterior release, posterior internal distraction, and subsequent posterior spinal fusion; PVCR, the posterior vertebral column resection and posterior spinal fusion; AVT, the apical vertebra translation.

*p < 0.1

## Discussion

In this study, all patients with severe and rigid scoliosis had a main curve of more than 80° on normal radiographs and the flexibility of less than 30% on bending radiographs. The incidence of distal adding-on was approximately 22.6% at a minimum 2-year follow-up, which is lower than that reported in the literature (3). For common AIS patients with a smaller curve, the incidence of distal adding-on has been reported to be 2%–13% (13). There are many hypotheses regarding the causes of distal adding-on in common AIS (14–17). However, severe and rigid scoliosis, as a special disease form, has a unique etiology, clinical manifestations and internal mechanisms that are different from those of AIS. Consequently, the mechanism and causes of the distal adding-on phenomenon have rarely been studied in depth.

Skeletal maturity is thought to be related to the occurrence of distal adding-on, and some researchers believe that lower skeletal maturity may also be associated (18). In this study, no obvious difference in the Risser grade was found, which may be because the patient reached bone maturity at the time of operation. Therefore, further research is needed to confirm this theory. Another factor to consider is the etiology of the patients, as treatment strategies may vary widely under different etiologies. Different etiologies can also lead to more confounding factors, complicating the study of distal adding-on in scoliosis. There are some related studies comparing scoliosis with different etiologies. A classic study by Sha et al. compared outcomes after spinal fusion surgery in syringomyelia-associated scoliosis (SMS) and AIS patients and found that despite the differences in preoperative status, AIS and SMS patients had comparable clinical and radiographic outcomes (19). Nevertheless, there is little data on distal adding-on in these patients. In this study, the etiology was not identified in severe and rigid scoliosis as a predictor of distal adding-on, which may mainly be limited by the sample size of syringomyelia-associated scoliosis and congenital scoliosis (CS).

According to our study, the correction rate of the primary curve was similar, and therefore distal adding-on caused by overcorrection was not observed. Notably, Post-AVT was associated with distal adding-on in the stepwise logistic regression analysis. As the degree of Post-AVT decreases, the probability of adding-on increases according to the nomogram. We hypothesize that as the degree of Post-AVT decreases, the body trunk requires greater compensatory force at the caudal side to maintain body balance. When this compensatory force exceeds a certain critical value, adding-on may appear, which may also explain why the CB and caudal disc angle in the Adding-on group were greater at the last follow-up. During the process of body trunk dynamic balance restoration, the CB and caudal disc angle may change to achieve somatic balance. Except for the cause of the caudal side, the shoulder balance needs to be observed because it is closely related to distal adding-on in AIS patients, especially in Lenke type II. In previous studies, distal adding-on was shown to play a positive role in maintaining shoulder balance (20). However, no significant correlation was observed in the parameters of shoulder balance, including CA, CHD, CRID, and RSH, between the groups with and without distal adding-on in this study. One possible explanation is that the relationship between the shoulder balance and distal adding-on is so weak that it is difficult to identify in severe and rigid scoliosis.

Another highly controversial issue is the relationship between LIV and LTV, LSTV, LEV, NV, and SV. Substantial evidence suggests that inappropriate LIV selection may lead to distal adding-on (15, 21). It is currently recognized that LIV should be extended to or beyond the LTV/LSTV to prevent distal adding-on. In most patients in our study, the instrumented levels were extended to or beyond the LTV/LSTV; therefore, relevant evidence still needs to be further explored.

**Figure 2:**
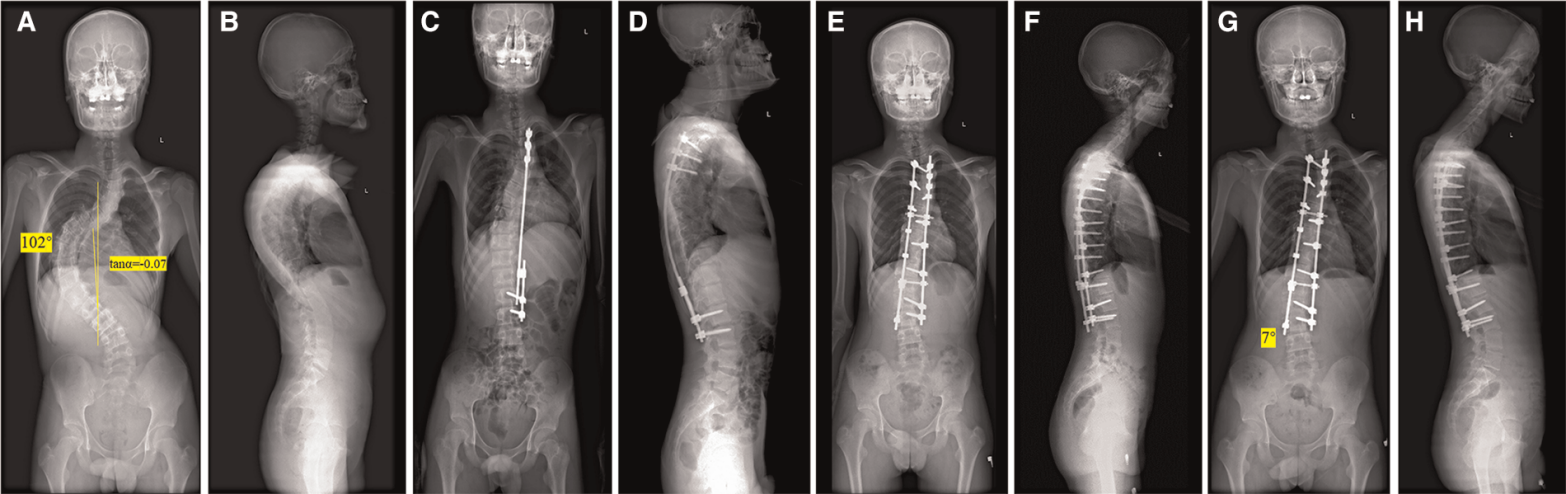
A 15-year-old boy with a severe and rigid thoracic curve (**A,B**). The radiographs demonstrate a thoracic curve angle of 102°, and the slope of the line connecting the pedicles on the concave side of the upper- and lower-end vertebrae (Tan *α*) is −0.07. After-distraction posteroanterior (**C**) and lateral (**D**) radiographs demonstrate well-maintained global coronal and sagittal balance. Postoperative posteroanterior (**E**) and lateral (**F**) radiographs following posterior spinal fusion from T2 to L3 demonstrate well-maintained global coronal and sagittal balance. Radiographs made at the final follow-up (**G,H**) reveal the appearance of the distal adding-on phenomenon with an intervertebral space angle below the LIV of 7°.

**Figure 3:**
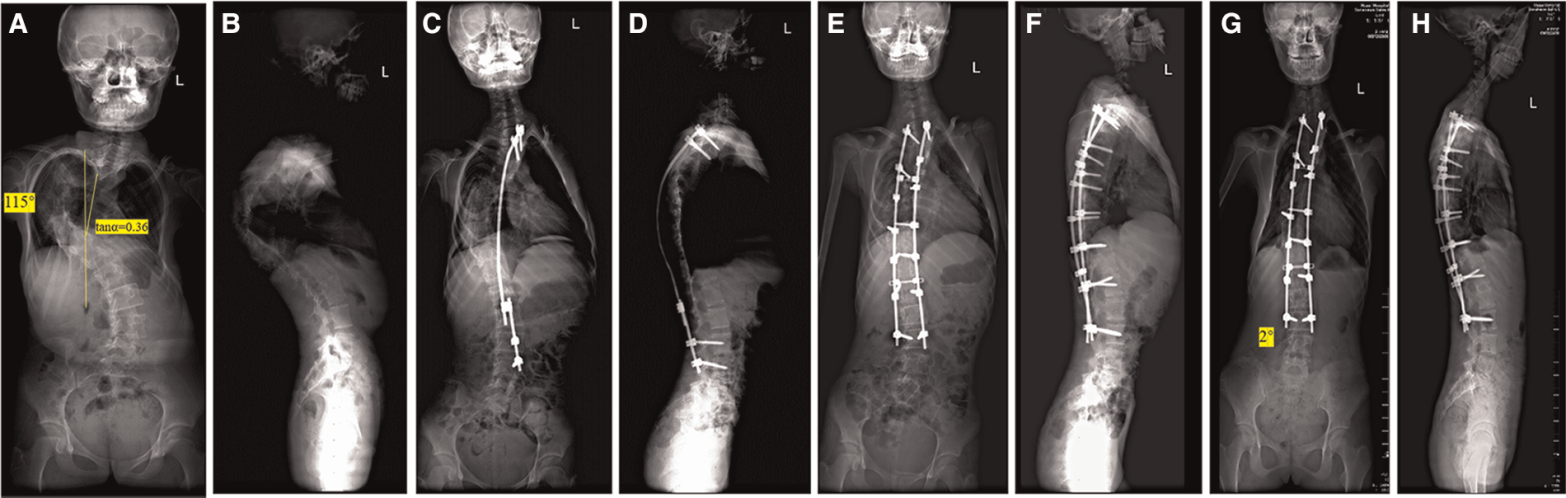
A 16-year-old girl with a severe and rigid thoracic curve (**A,B**). The radiographs demonstrate a thoracic curve angle of 115°, and the slope of the line connecting the pedicles on the concave side of the upper- and lower-end vertebrae (Tan *α*) is 0.36. After-distraction posteroanterior (**C**) and lateral (**D**) radiographs demonstrate well-maintained global coronal and sagittal balance. Postoperative posteroanterior (**E**) and lateral (**F**) radiographs following posterior spinal fusion from T2 to L3 demonstrate well-maintained global coronal and sagittal balance. Radiographs made at the final follow-up (**G,H**) reveal well-maintained global coronal and sagittal balance with absence of the distal adding-on phenomenon.

Notably, the preoperative Tan *α* in severe and rigid scoliosis was found to be related to distal adding-on. According to the nomogram, as the preoperative Tan *α* decreased from positive to negative values, the probability of distal adding-on increased. This phenomenon shares many similarities with the previously proposed S-line in Lenke type 5C but differs in its definition and essence (22). In Lenke type 5C AIS patients, spine surgeons change the S-line from a positive to a negative condition with one of a number of techniques to avoid postoperative coronal decompensation. This demonstrates that connecting lines such as the S-line play a role in predicting or reflecting the state of body trunk dynamic balance restoration. Similarly, the preoperative Tan *α* predicted distal adding-on at the last follow-up. Our data include a 15-year-old boy and the radiographs demonstrated a thoracic curve of 102° and a Tan *α* of −0.07. Radiographs made 2 years after surgery revealed the appearance of the distal adding-on phenomenon with an intervertebral space angle below the LIV of 7° (Figure 2). In 16-year-old girl and the radiographs demonstrated a thoracic curve of 115° and a Tan *α* of 0.36). Two years after surgery, radiographs made obtained well-maintained global coronal and sagittal balance with the absence of distal adding on (Figure 3). We hypothesize that as the preoperative Tan *α* decreases from positive to negative, the stress area maintaining body trunk balance on the caudal side moves down, and under similar instrumented levels, the stress exceeds a certain acceptable critical value, decompensation occurs in the body trunk, and distal adding-on soon develops.

Among the three surgical approaches, the IP procedure demonstrated the best effect on preventing distal adding-on, followed by PVCR; APSF was the most likely to increase the probability of distal adding-on. Compared with APSF, IP and PVCR can release the internal stress of the spine to a greater extent and thus largely prevent the occurrence of distal adding-on. We combined the indications for distal adding on to create a nomogram, whose results suggest that when the preoperative Tan *α* is a negative value, spine surgeons should select the appropriate LIV and prioritize IP and PVCR for preventing the occurrence of distal adding-on. And during the correction process, a smaller Post-AVT should not be overly pursued.

This study has several limitations. First, it was a single-center study performed retrospectively. Second, patients with main lumbar curvature were excluded from this study; therefore, not all types of severe and rigid scoliosis were analyzed. Finally, there were no related data on the Research Society-22 score for evaluating the clinical outcome. Finally, the patients in the data set possessed their own characteristics, and the treatment methods also involved the characteristics of the center. Therefore, whether it is effective in other larger data sets still needs further verification and further research should be conducted to optimize these deficiencies.

## Conclusion

In conclusion, by combining associated factors of distal adding-on in severe and rigid scoliosis patients, a nomogram was constructed. The operation, Post-AVT and Tan α were identified as predictors of distal adding-on. For patient with a negative Tanα in severe and rigid scoliosis, the risk of distal adding-on tended to increase, and it is recommended to give priority to IP or PVCR. In the final correction, a smaller Post-AVT should not be pursued excessively.

## Data Availability

The original contributions presented in the study are included in the article/Supplementary Material, further inquiries can be directed to the corresponding author/s.
